# Defence-related metabolic changes in wheat (*Triticum aestivum* L.) seedlings in response to infection by *Puccinia graminis* f. sp. *tritici*

**DOI:** 10.3389/fpls.2023.1166813

**Published:** 2023-06-12

**Authors:** Mercy Maserumule, Molemi Rauwane, Ntakadzeni E. Madala, Efficient Ncube, Sandiswa Figlan

**Affiliations:** ^1^Department of Agriculture and Animal Health, School of Agriculture and Life Sciences, College of Agriculture and Environmental Sciences, University of South Africa, Roodepoort, South Africa; ^2^Department of Botany, Nelson Mandela University, South Campus, Port Elizabeth, South Africa; ^3^Department of Biochemistry and Microbiology, Faculty of Sciences, Agriculture and Engineering, University of Venda, Thohoyandou, Limpopo, South Africa

**Keywords:** LC-MS, stem rust, *P. graminis* f. sp. *tritici*, primary and secondary metabolism, wheat metabolomics

## Abstract

Stem rust caused by the pathogen *Puccinia graminis* f. sp. *tritici* is a destructive fungal disease-causing major grain yield losses in wheat. Therefore, understanding the plant defence regulation and function in response to the pathogen attack is required. As such, an untargeted LC-MS-based metabolomics approach was employed as a tool to dissect and understand the biochemical responses of Koonap (resistant) and Morocco (susceptible) wheat varieties infected with two different races of *P. graminis* (2SA88 [TTKSF] and 2SA107 [PTKST]). Data was generated from the infected and non-infected control plants harvested at 14- and 21- days post-inoculation (dpi), with 3 biological replicates per sample under a controlled environment. Chemo-metric tools such as principal component analysis (PCA), orthogonal projection to latent structures-discriminant analysis (OPLS-DA) were used to highlight the metabolic changes using LC-MS data of the methanolic extracts generated from the two wheat varieties. Molecular networking in Global Natural Product Social (GNPS) was further used to analyse biological networks between the perturbed metabolites. PCA and OPLS-DA analysis showed cluster separations between the varieties, infection races and the time-points. Distinct biochemical changes were also observed between the races and time-points. Metabolites were identified and classified using base peak intensities (BPI) and single ion extracted chromatograms from samples, and the most affected metabolites included flavonoids, carboxylic acids and alkaloids. Network analysis also showed high expression of metabolites from thiamine and glyoxylate, such as flavonoid glycosides, suggesting multi-faceted defence response strategy by understudied wheat varieties towards *P. graminis* pathogen infection. Overall, the study provided the insights of the biochemical changes in the expression of wheat metabolites in response to stem rust infection.

## 1 Introduction

Wheat (*Triticum aestivum* L) is an important grain crop contributing 40% of the calorie intake and supporting 35% of the food intake of the global population (Grote et al., 2021). In the African continent, wheat has become one of the important staple food crops due to rapid population increases and changes in food habits. According to FAO (2017), Africa produces more than 25 million tons (MT) of wheat on 10 million hectares (Mha) of land, where sub-Saharan Africa (SSA) accounts for 40% of the produced wheat with 7.5 MT on a total area of 2.9 Mha. The most important wheat producing countries in SSA are Ethiopia, South Africa, Sudan, Kenya, Tanzania, Nigeria, Zimbabwe and Zambia (FAO, 2017). Ethiopia has the largest production at 1.7 Mha, followed by South Africa at 1.5 Mha. The production in the continent is regrettably not sufficient to meet the demands. As a result, from 2013 to 2019, African countries imported 16.9 MT of wheat at a cost of USD 6 billion, which exhaust inadequate foreign currency reserves of respective countries (FAO/WHO Human vitamin and mineral requirements, 2020).

According to Tadesse et al. (2018), the growing human population, rural-urban migration, inappropriate new agricultural policies and low adoption rates of new technologies remain major challenges for wheat production in developing countries. In addition, wheat production is constantly facing challenges such as climate change, increased inputs costs, abiotic (drought and heat) and biotic (diseases and pests) stresses. Among the biotic stresses, the most common diseases of wheat include wheat rusts, Fusarium head blight caused by *Fusarium graminearum* and powdery mildew caused by *Blumeria graminis* f. sp. *tritici* (Duveiller et al., 2007; Mondal et al., 2016; Deihimfard et al., 2022). Wheat rust such as stem rust caused by *Puccinia graminis* f. sp. *tritici* (*Pgt*), leaf rust (*P. triticina* Eriks. - *Pt*) and stripe rust (*P. striiformis* f. sp. *tritici - Pst*) are amongst the most destructive diseases of the crop. Great yield losses have been experienced worldwide, and these rust pathogens continue to evolve and threaten wheat production globally (Boshoff et al., 2018; Saunders et al., 2019; Davis et al., 2020; Li et al., 2022).

The emergence of a highly destructive wheat stem rust race, Ug99 (TTKSK) in Uganda in 1998 has threatened the global wheat production. A series of reviews by Singh et al. (2006); Singh et al. (2008); Singh et al. (2011); and Singh et al. (2015) have reported the significance, emergence, evolution and geographical spread of the Ug99 group. To date, there’s a record of 15 known variants within the Ug99 lineage which have been identified in 14 countries (https://rusttracker.cimmyt.org/?page_id=22). South Africa was included on the Ug99 list in 2000, with currently five races of Ug99 present in the country (Terefe et al., 2019). The races include TTKSF (2SA88; Pretorius et al., 2000), TTKSP (2SA106; Pretorius et al., 2007), PTKST (2SA107; Visser et al., 2011), TTKSF+ (2SA88+; Visser et al., 2011) and PTKSK (2SA42; Terefe et al., 2021). These races vary from one another through virulence profiles against different wheat varieties with different resistant genes (Zhao et al., 2019). TTKSF and TTKSP are amongst the races that virulence was confirmed on wheat varieties that are known to possess the rust resistance genes such as *Sr31*, *Sr24* and *Sr36* that are now ineffective against the related races of Ug99 (Mondal et al., 2016). Thus, these studies highlighted the evolving pathogen potential and the need to develop stem rust resistant wheat varieties that will in turn boost productivity.

Improved host plant resistance is the most profitable and environmentally friendly control strategy to mitigate biotic stress (Kloppers and Pretorius, 1997; McCallum et al., 2016). Progress in understanding the underlying biochemical and molecular basis of rust diseases in wheat will facilitate resistance breeding through the use of biotechnology approaches. Omics studies such as metabolomics can assist in unravelling and better understanding metabolic responses of wheat to biotic stress for breeding programmes. Additionally, a combination of metabolomics and other omics studies may/can lead to the development of biomarkers for resistance/tolerance checks. In previous studies on oat (*Avena sativa*) (Li et al., 2022), sorghum (*Sorghum bicolor*) (Tugizimana et al., 2019), wheat (*Triticum aestivum* L), common bean (*Phaseolus vulgaris* L) (Makhumbila et al., 2023) among others, metabolomics has been used for comparative metabolomic phenotyping to identify biomarkers or metabolic signatures linked to varied response capacities shown in varieties with varied resistance potentials. Therefore, identifying biochemical changes of primary and especially secondary metabolites need to be considered when observing plant-pathogen interactions. Hence, this study aimed to identify the metabolomic changes of two wheat varieties (Koonap and Morocco) in response to infection by rust pathogen *P. graminis* at different time-points, using an untargeted LC-MS metabolomic approach.

## 2 Materials and methods

### 2.1 Plant and pathogen material

Seeds of two wheat varieties (Koonap and Morocco) used in this study were obtained from the Agricultural Research Council-Small Grain Institute (ARC-SGI, South Africa) germplasm bank (**Table 1**). The rust isolates (2SA88 and 2SA107) were also obtained from ARC-SGI for inoculation purposes.

**Table 1:** Wheat varieties selected for stem rust screening.

Cultivar	Origin	Pedigree	Characteristic
Koonap	SGI (2010)	IP rights	Intermediate type, medium to high yield potential, medium to high growth length, excellent straw strength
Morocco*	Obscure; considered a North African cultivar	-	-

*Morocco (universal susceptible); IP, Intellectual Property.

### 2.2 Planting and inoculation

Wheat seeds (25 per pot) were sown in plastic pots of 10 cm diameter filled with steam-sterilised soil. Prior to planting, the soil was treated with water soluble fertiliser (10 g L^-1^) containing nitrogen (15%), phosphorus (4.5%) and potassium (26.3%). After emergence, plants were fertilised twice with 10 g L^-1^ multi-feed (Nulandis, South Africa) water soluble fertiliser (19:8:16 NPK plus micronutrients). Seedlings were grown under light for 6 to 7 days in a temperature-controlled seedling room (22-25 °C) and a rust-free environment prior inoculation.

For inoculation, urediniospores of stem rust isolates (2SA88 and 2SA107) stored at -80°C were heat-shocked in lukewarm water (about 40°C) for 10 minutes. Urediniospores were suspended in light mineral oil (Soltrol 170: Chevron Phillips, United State of America) at a concentration of 5 mg spores/mL (6x106 spores/mL) and sprayed onto the fully expanded primary leaves of wheat seedlings. For mock inoculation, Soltrol 170 (without the rust isolates) was also sprayed on control plants (Pretorius et al., 2000; Woldeab et al., 2017). Seedlings were incubated at 18°C in a dew chamber with relative humidity of 43% for 16 hours. Upon removal from the chamber, plants were exposed under fluorescent light for 3 hours. Inoculated plants were then placed in a greenhouse at a minimum temperature of 15°C and maximum of 25°C. Separate compartments in a greenhouse were used for different treatments. Each experiment was replicated 3 times, three different pots per variety were used to grow three biological replicates of each variety.

### 2.3 Disease scoring

Wheat seedlings were evaluated phenotypically and scored on a scale of 0 to 4 according to Stakman et al. (1962). Symptom severity was evaluated per plant whereby the scale of 0 - 2 represents low infection type, scale 3 – 4 represents high infection type. The symbols ‘;’ representing macroscopic hypersensitive flecks, ‘X’ representing a mesothetic or mixed reaction, and ‘+’ and ‘-’ indicating ‘more’ or ‘less’, respectively, were also used. Infection recordings were taken at 14 days and 21 days post-inoculation (dpi).

### 2.4 Extraction and quantification of leaf tissue material

Metabolite extraction was carried out according to the modified version of Madala et al. (2014). One gram (1g) of each leaf sample was ground into powder with liquid nitrogen using a mortar and pestle. The sample was resuspended in 1.5 ml of 80% ice-cold methanol in a 2 ml centrifuge tube and vortexed for 30 sec. The sample was subjected to sonication for 30 min and centrifuged at 5000 ref per minute (rpm) for 5 min at 4 °C. The supernatant (extract) was transferred to a new 2 ml Eppendorf tube and stored at 4 °C for further analysis.

### 2.5 LC-qTOF-MS analysis

**Figure 1:**
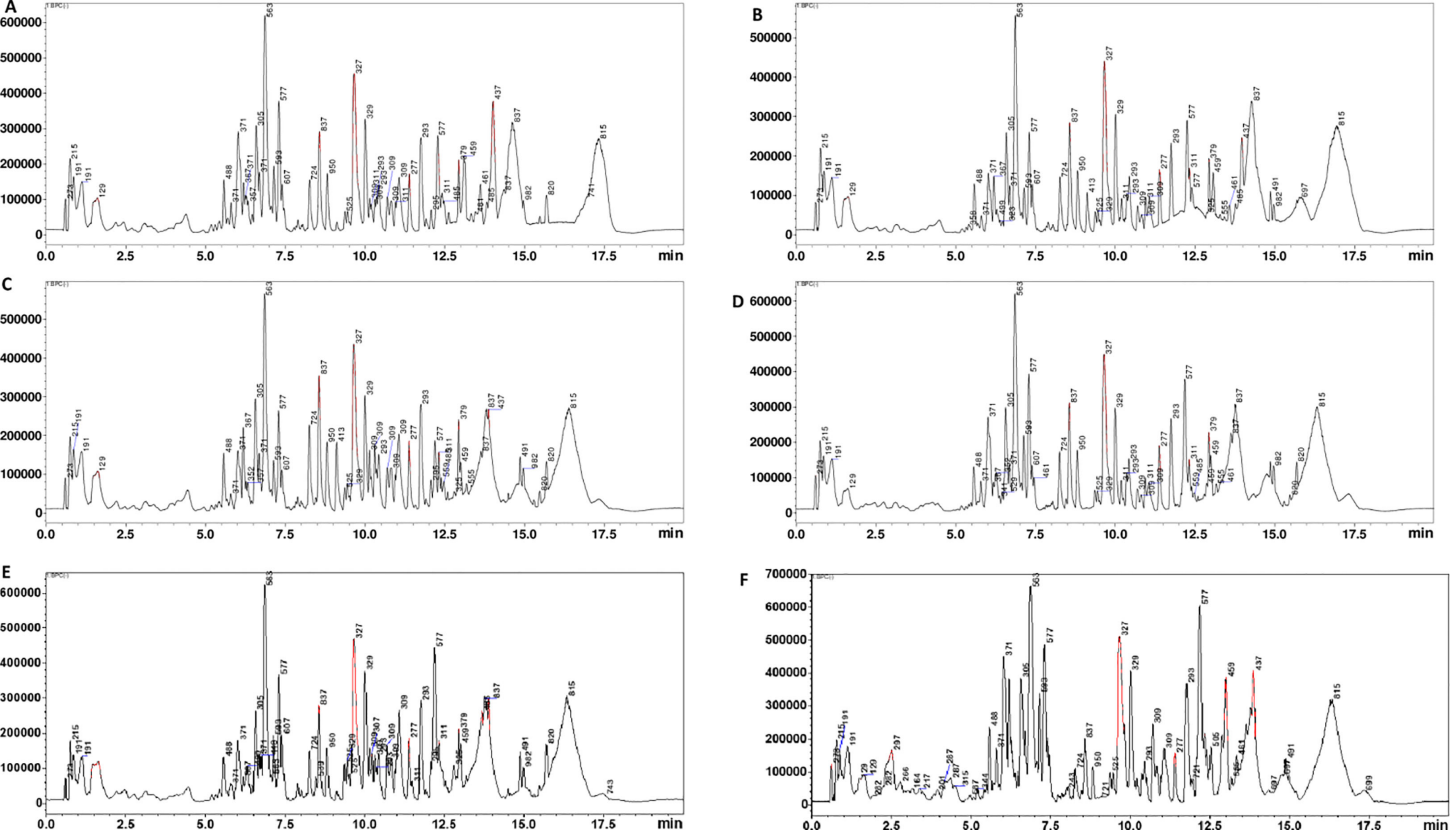
Representative UHPLC-QTOF-MS base peak intensity (BPI) chromatograms of Koonap cultivar methanolic-extracts. BPI MS chromatograms revealed differentially populated peaks for Koonap infected with rust race 2SA88 at 14- and 21- dpi (**A, B**, respectively), infected with rust race 2SA107 at 14- and 21 dpi (**C, D**, respectively) and control (**E, F**, respectively) each with unique m/z values, intensities and retention times (Rt’s), representing the qualitative (presence/absence) and quantitative (intensity/concentration) detection of metabolites, thus providing a visual description of the similarities and differences between the selected wheat varieties.

Samples were filtered with 0.22 µm nylon filters attached to 500 µL inserts (Thermo Fisher, Johannesburg, South Africa) to sieve the plant extract and transferred into HPLC glass vials. Plant extracts were analysed on liquid chromatography-quadrupole time-of flights tandem mass spectrometry instrument (LCMS-9030 qTOF, Shimadzu Corporation, Kyoto, Japan) for separation and detection of metabolites as described by Ramabulana et al. (2021). The chromatographic separation was performed on a Shim-pack Velox C18 column (100 mm x 2.1 mm with a particle size of 2.7 µm) (Shimadzu Corporation, Kyoto, Japan) housed inside a column oven set at 55°C. An injection volume of 3 µL was used for all samples, and a binary solvent system consisting of solvent A: 0.1% formic acid in Milli-Q water (both HPLC grade, Merck, Darmstadt, Germany) and solvent B: Methanol (UHPLC grade, Romil SpS, Cambridge, United Kingdom) with 0.1% formic acid pumped at a constant flow rate of 0.4 mL/min. A 20 min multiple gradient method was used to achieve the separation of metabolites. The starting condition were isocratic for 5% B for 3 min which was followed by gradual increase to 40% B for 2 min and later to 95% B for 7 min and kept isocratic at 95% B for 4 min, the conditions were then returned to 5% B in 2 min and kept constant for another 2 min at 5% B to re-equilibrate the column for the next injection. Chromatographic elution was monitored using qTOF high-definition mass spectrometer that was set to negative electrospray ionisation mode using data dependant acquisition (DDA) mode. Here, metabolic profiling was carried out in the negative electron ionization (ESI) mode (**Figure 1**), in order to better ionize phenolic compounds (such as flavonoids and carboxylic acids) (Hamany Djande et al., 2021). The subsequent parameters were set as follows: interface voltage (4.0 kV), interface temperature (300°C), nebulization and dry gas flow (3 L/min), heat block (400°C), DL temperature (280°C), detector voltage (1.8 kV), and flight tube temperature (42°C). Ion fragmentation was achieved using argon gas for collision induced dissociation (CID) experiments with energy of 30eV and 5 eV spread. NaI was used as a calibrant to ensure mass accuracy below 1 ppm.

### 2.6 Analysis of metabolites data

Extracts were converted to mzML files on Lab solutions (Shim-pack UFLC SHIMADZU CBM20A). The acquired raw datasets were processed using XCMS Online (http://XCMSOnline.scripps.edu/) (accessed on 10 July 2022). The data was processed with the following HPLC/UHD-qTOF parameters: feature detection was performed using the cent Wave method, maximal tolerated m/z was set to 30 ppm, signal-to-noise ratio was set to 10, the prefilter intensity and noise filter were set to 700 and 15, respectively. The retention time correction was performed using the ordered bijective interpolated warping (OBI-Warp method with a profStep of 0.4. Other parameters were set as bandwith = 0.5, minfrac (minimum fraction of sample in a group to be referred to as a feature) and mzwid (m/z width to determine peak groupings) of 0.020. The Kruskal–Wallis non-parametric method was used to perform the statistical test, *post-hoc* analysis was also performed, and the data was normalized using the median fold change. The resulting feature table with 2382 features was imported into SIMCA (soft independent modelling of class analogy) version 17.0 software (Sartorius, South Africa). The imported data were scaled using standard deviation by applying Pareto-scaling (Van den Berg et al., 2006). The models presented in this study are principal component analysis (PCA), Orthogonal projection to latent structures-discriminant analysis (OPLS–DA) and hierarchical cluster analysis (HCA). These are exploratory unsupervised models that assess the structure of a dataset highlighting trends or patterns within a dataset (Tugizimana et al., 2020). The explanation and description of these tools (PCA, OPLS-DA and HCA) have been detailed in literature (Saccenti et al., 2013; Saccenti et al., 2018; Saccenti et al., 2019). To complement the descriptive view provided by PCA modelling, orthogonal projection to latent structures-discriminant analysis (OPLS-DA) modelling of treated vs non-treated control samples was performed. The respective S-plots of the OPLS-DA score plots were also generated.

Metabolite annotation and identification were concomitantly carried out using the BPI, and single ion extracted chromatograms from Koonap and Morocco varieties. Chemo-metrically extracted metabolites were used to perform metabolic pathway analysis using MetaboAnalyst Pathway Analysis (MetPA) to reveal impactful metabolic pathways associated with the generated data across the two varieties. To inspect induction of biological events at the metabolic pathway level under stem rust infection, a KEGG pathway enrichment test was carried out. Pathway enrichment was used to identify the metabolic pathways to which the significantly changed metabolites are associated by mapping our metabolites data with the KEGG database.

## 3 Results

### 3.1 Phenotypic evaluation and scoring

**Figure 2:**
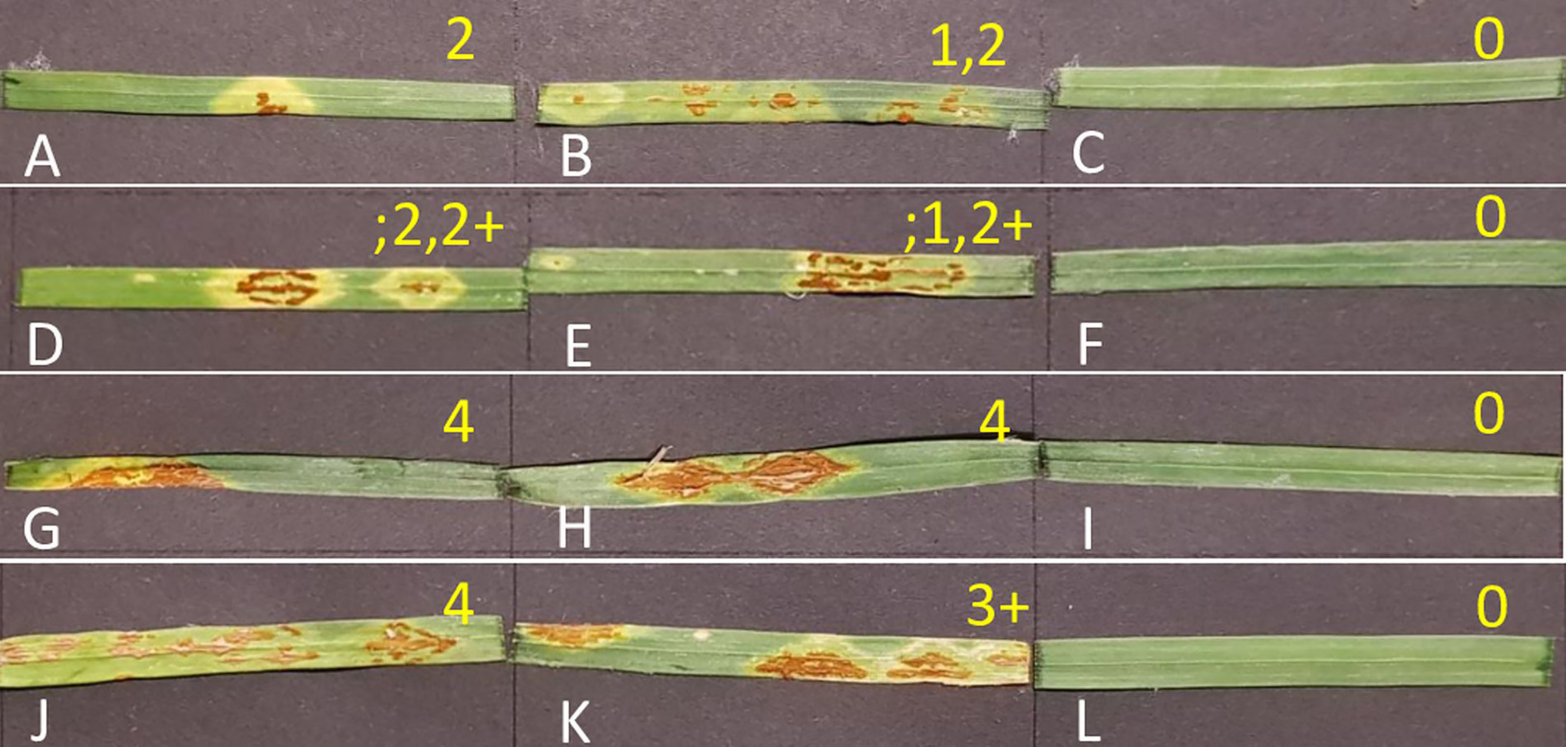
Stem rust response of Koonap and Morocco seedlings at different time-points. Koonap infected with rust races 2SA88, 2SA107 and control at 14dpi (**A–C**, respectively) and 21- dpi (**D–F**, respectively). Morocco infected with rust races 2SA88, 2SA107 and control at 14 dpi (**G–I**, respectively) and 21 dpi (**J–L**, respectively). Effective rust infection can be observed on the 2SA88 and 2SA107 treated plants relative to the control plants, where the appearance of the flecks (yellowing) and the spores (browning) appeared from 14 dpi and progressed (lesion elongation) over the days to 21 dpi. Infection types used to score wheat seedlings from 14 to 21 dpi. 0= immune,;= flecks, 1= minute uredinia, 2= small to medium sized uredinia, 3= large uredinia encircled by chlorosis, 4= large uredenia usually without any chlorosis.

The phenotypic evaluation and scoring of Koonap and Morocco varieties showed distinct differences between the infected and non-infected plants at different time-points (**Figure 2**). Koonap (resistant) showed low infection scores (0 – 2) for all three experiments at 14- and 21- dpi for the races as shown on **Figure 2A** (2SA88 at 14dpi) and **2B** (2SA107 at 14dpi) as well as **Figure 2D** (2SA88 at 21dpi) and **2E** (2SA107 at 21dpi). On the contrary, Morocco (susceptible) showed high infection scores (3 – 4) for both races at 14- and 21- dpi as shown on **Figure 2G** (2SA88 at 14dpi) and **2H** (2SA107 at 14dpi) and **2J** (2SA88 at 21dpi) and **2K** (2SA107 at 21dpi), respectively. **Figures 2C, F, I, L** are the non-infected controls for both varieties, showing no symptoms of disease for both races at different time-points.

### 3.2 LC-qTOF-MS-analysis

Extracted LC chromatograms with distinct base peak intensities (BPI) revealed good chromatographic separation [**Figure 1** (Koonap) and **Supplementary Figure S1** (Morocco)]. The visual inspection of the extracted LC chromatograms displaying distinct BPI from ESI negative data shows chromatographic separation with slight or minor differences in some peak intensities of the metabolomes. The LC-qTOF-MS data sets were further analysed with explorative as well as predictive multivariate analyses to highlight the metabolic changes.

### 3.3 Multivariate data analysis

**Figure 3:**
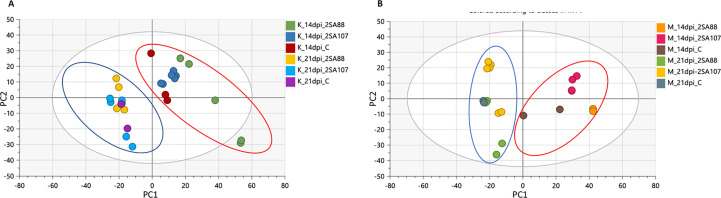
Unsupervised exploratory data analysis of ESI negative wheat cultivars classification data. Principal component analysis shows the grouping of *Pgt*-resistant Koonap cultivar **(A)**, while the susceptible Morocco cultivar **(B)** shows discriminant sample clustering coloured by time-point and race. The PCA scatter plots displayed a time-dependant response to infection. The diagnostic parameters of the score plot, generated from PC1 and PC2, are as follows; R^2^ X (cum) = 88%, Q^2^ (cum) = 73%.

The PCA score scatterplot for Koonap and Morocco (**Figures 3A, B**, respectively) revealed differential clustering between sample groups based on the races 2SA88, 2SA107 and the non-treated control at different time-points. The sample clustering was according to the 14- and 21- dpi time-points (as shown by blue and red circles on **Figure 3**), which is indicative of the different metabolite profile concentrations as the disease progressed. Also noteworthy, the differential clustering of the varieties reflected both differential quantitative distribution of each metabolite among the varieties as well as the qualitative makeup of metabolites.

**Figure 4:**
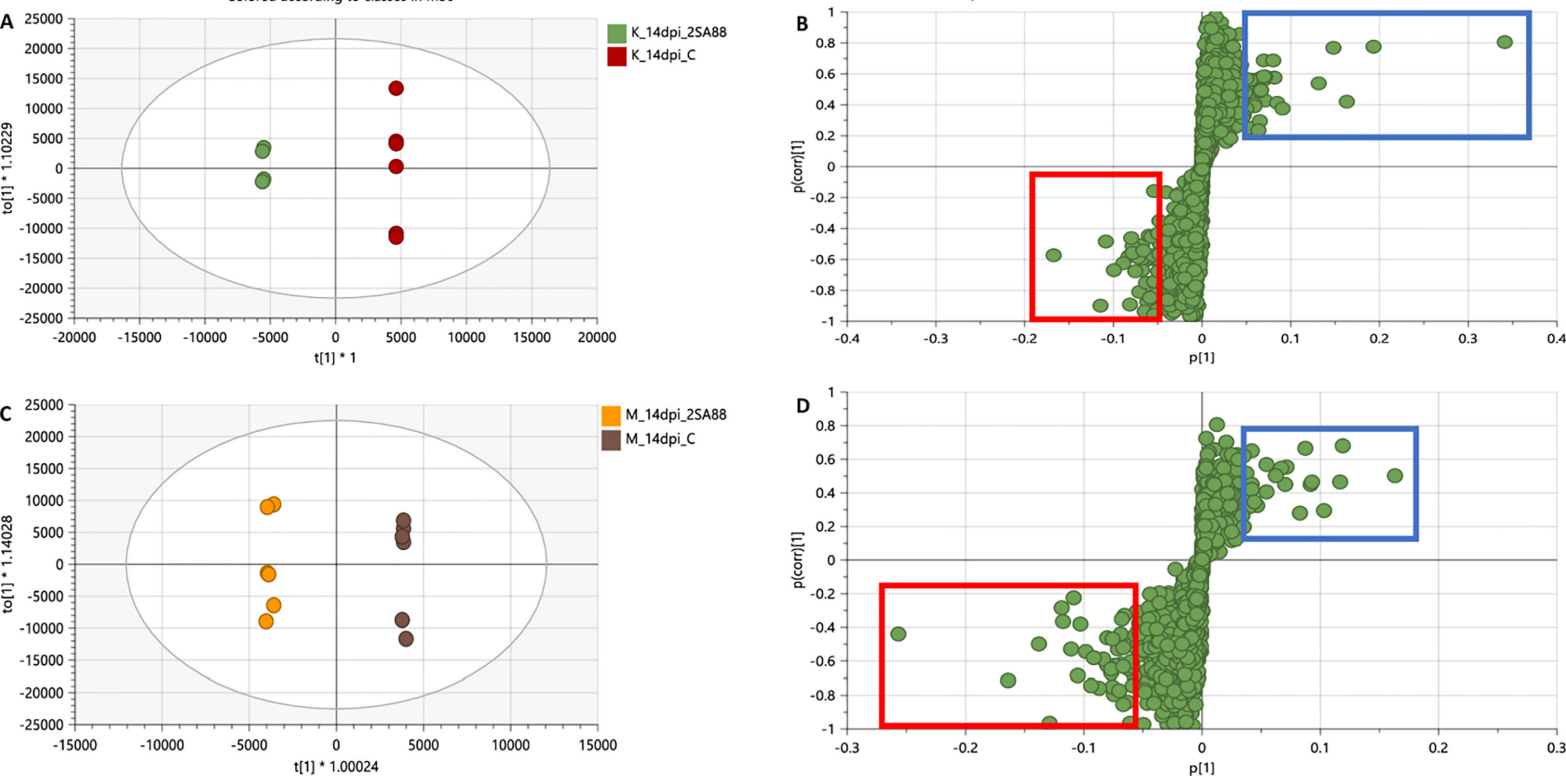
OPLS-DA modelling showing the selection of signatory biomarkers associated with response of rust race infections of 2SA88 and 2SA107. The OPLS-DA scores plot **(A, C)** showed clear discrimination between the treated samples and control whereas S-plots **(B, D)** allowed for the extraction of significant biomarkers. Extracted ions representing significant biomarkers responsible for the difference between the control at the top right corner (in the blue rectangle) and samples treated with *Pgt*. at the bottom left corner (in the red rectangle) for both Koonap and Morocco cultivars, respectively. The loading vector of the predictive component is on the y-axis, while the x-axis represents the modelled covariation.

The OPLS-DA further confirmed that the metabolite profiles in varieties were differentially regulated over time as evidenced in **Figures 4**, **S2**. This simply indicated that the data structures extracted by OPLS-DA modelling point to underlying differences in the measured metabolite profiles in the different sample groups. This observation was then further investigated by applying tools for annotating metabolites and performing quantitative assessments as shown and discussed below. The biomarkers as shown on the S-plots were also putatively annotated.

### 3.4 Metabolite annotation

**Figure 5:**
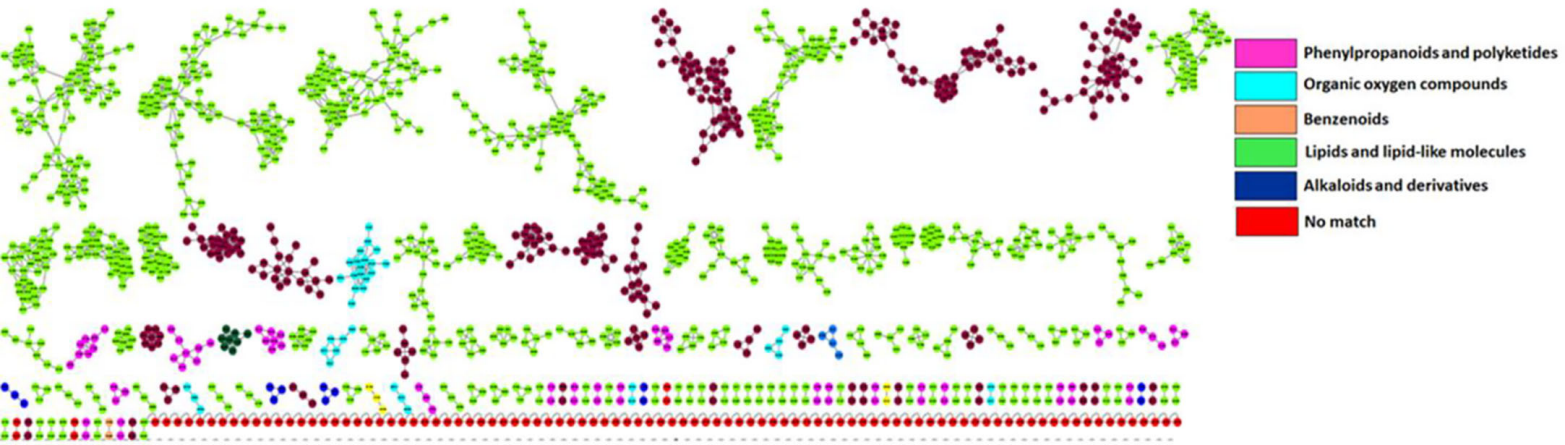
Molecular network of LC-MS spectra generated with MolNetEnhancer (in GNPS) giving a metabolome coverage and classes of extracted metabolites from *Pgt* treated and non-treated control plants. Each displayed node represents a metabolite, while each cluster of pooled nodes (coloured) depicts a class of chemically related and putatively annotated metabolites matched to GNPS libraries and databases. Red nodes represent unmatched spectral data.

Putative annotation of metabolites led to the identification of 51 metabolites of different classes originating from both primary and secondary metabolic pathways. A profile of these metabolites is illustrated in **Table 2**, listing the individual annotated metabolites commonly expressed within the two varieties or present as unique biomarkers. Metabolite annotation was performed to level 2 of the Metabolomics Standard Initiative (MSI) based on product ion spectral information formed by collision-induced dissociation (CID) of selected parent ions (Sumner et al., 2007). Molecular networking was used to confirm the classes of positively identified metabolites (**Figure 5**) and of these, significant classes are further discussed below.

**Table 2:** Summary of the putatively annotated metabolites from Koonap and Morocco wheat cultivars, untreated P(UT) and treated (PT), infected with 2SA88 and 2SA107 stem rust races at 14- and 21- dpi.

Metabolite	Experimental *m/z*	MW	MF	Rt	K14dpi 2SA88	K21dpi 2SA88	K14dpi 2SA107	K21dpi 2SA107	M14dpi 2SA88	M21dpi 2SA88	M14dpi 2SA107	M21dpi 2SA107	Biological role
Canrenone	339,2	340,5	C**_22_** H**_28_** O**_3_**	15,51		P(T)							Flavanoid
Vicenin-3 I	563,13	564,5	C**_26_** H**_28_** O**_14_**	7,35	P(UT)		P(UT)			P(UT)			Flavanoid
Guanosine	282,08	283,24	C**_10_** H**_13_** N**_5_** O**_5_**	2,05							P(T)		Hormone
2,6-Di-tert-butyl-4-nitrophenol	250,14	251,32	C**_14_** H**_21_** NO**_3_**	11,96									Flavonoid
9,12,15-Octadecatrienoic acid, 3-(hexopyranosyloxy)-2-hydroxypropyl ester, (9Z,12Z,15Z)-	512,31	514,6	C**_27_** H**_46_** O**_9_**	12,25									Fatty acid human
Canrenone	339,2	340,5	C**_22_** H**_28_** O**_3_**	13,28									Flavonoid
Vicenin-2 I	593,15	594,5	C**_27_** H**_30_** O**_15_**	7,07	P (UT)		P(UT)	P (T)					Flavanoid
(2E)-3-[4-({2-O-[(2S,3R,4R)-3,4-Dihydroxy-4-(hydroxymethyl)tetrahydro-2-furanyl]-beta-D-glucopyranosyl}oxy)-3-methoxyphenyl]acrylic acid	487,15	488,4	C**_21_** H**_28_** O**_13_**	2,72									Hormone
Vicenin-3 II	563,18	564,5	C**_26_** H**_28_** O**_14_**	6,86									Flavanoid
Vitexin-2-rhamnoside	577,15	578,5	C**_27_** H**_30_** O**_4_**	7,29	P(UT)		P(UT)	P (T)		P(UT)	P(T)	P(UT)	Flavanoid
Chrysoeriol	299,06	300,26	C**_16_** H**_12_** O**_6_**	11,08									Flavanoid
[(4E)-7-acetyloxy-6-hydroxy-2-methyl-10-oxo-2,3,6,7,8,9-hexahydrooxecin-3-yl] (E)-but-2-enoate	325,13	326,34	C**_16_** H**_22_** O**_7_**	12,75		P(T)			P(T)		P(T)		Flavanoid
Saponarin I	593,15	594,5	C**_27_** H**_30_** O**_15_**	6,77									Flavanoid
Orientin	447,09	448,4	C**_21_** H**_20_** O**_11_**	12,31									Flavanoid
Decylbenzenesulfonic acid	297,15	298,4	C**_16_** H**_26_** O**_3S_**	11,76	P(UT)				P(T)	P(UT)		P(UT)	Flavonoid
(10E,15E)-9,12,13-trihydroxyoctadeca-10,15-dienoic acid	327,42	328,4	C**_18_** H**_32_** O**_5_**	14,03				P (T)					Fatty acid
Andrastin A	485,28	486,6	C**_28_** H**_38_** 0**_7_**	6,64				P (T)					Hormone
Glc-Glc-octadecatrienoyl-sn-glycerol	675,36	676,4	C**_33_** H**_56_** O**_14_**	13,34	P (T)	P (T)	P (T)		P(T)			P(T)	Lipids
Apigenin-6-C-glucoside-7-O-glucoside	593,15	594,5	C**_27_** H**_30_** O**_15_**	10,93						P(UT)		P(UT)	Flavanoid
4aR,5S,8aS,9aR)-9a-hydroxy-3,4a,5-trimethyl-5,6,7,8,8a,9-hexahydro-4H-benzo[f][1]benzofuran-2-one	249,11	250,33	C**_15_** H**_22_** O**_3_**	10,15									Flavanoid
Thymol-beta-D-glucoside	311,11	312,4	C**_16_** H**_24_** 0**_6_**	12,3				P (T)		P(T)		P(T)	Flavanoid
[6-[3,4-dihydroxy-2,5-bis(hydroxymethyl)oxolan-2-yl]oxy-3,4,5-trihydroxyoxan-2-yl]methyl (E)-3-(4-hydroxy-3-methoxyphenyl)prop-2-enoate	517,15	518,5	C**_22_** H**_30_** O**_14_**	6,57									Flavanoid
[5-hydroxy-6-[2-(4-hydroxy-3-methoxyphenyl)ethoxy]-2-(hydroxymethyl)-4-(3,4,5-trihydroxy-6-methyloxan-2-yl)oxyoxan-3-yl] (E)-3-(4-hydroxy-3-methoxyphenyl)prop-2-e	637,22	638,6	C**_30_** H**_30_** O**_15_**	14,18	P (T)	P (T)	P(UT)	P (T)	P (T)	P(UT)			Flavanoid
Octyl-methoxycinnamate	290,4	291,16	C**_18_** H**_26_** O**_3_**	11,76									Flavanoid
1-[2-methyl-6-[(2S,3R,4S,5S,6R)-3,4,5-trihydroxy-6-(hydroxymethyl)oxan-2-yl]oxyphenyl]ethanone	297,11	298,29	C**_14_** H**_18_** O**_8_**	10,19		P(T)		P(UT)				P(T)	Flavanoid
9-OxoODE	293,18	294,4	C**_18_** H**_30_** O**_3_**	12,75				P(UT)					Fatty acids
9-HOTrE	293,18	294,4	C**_18_** H**_30_** O**_3_**	10,82	P (UT)	P (UT)		P(UT)					Fatty acids
PG(16:0/18:3)	743,48	744,56	C**_40_** H**_73_** O**_10_** P	12,63	P (T)			P(UT)	P(UT)	P(UT)			Lipids
Norgestrel	311,19	312,4	C**_21_** H**_28_** O**_2_**	12,18					P(T)				Flavanoid
2’-Deoxyguanosine	266,09	267,2	C**_10_** H**_13_** N**_5O4_**	11,65	P(UT)		P(UT)		P(T)				Hormone
2-(4-Methyl-3-cyclohexen-1-yl)-2-propanyl 6-O-(6-deoxy-alpha-L-mannopyranosyl)-beta-D-glucopyranoside	461,2	462,5	C**_22_** H**_38_** O**_10_**	13,56									Flavonoid
Dictamnoside D	449,28	450,5	C**_21_** H**_38_** 0**_11_**	13,08									Flavonoid
(3S)-5-[(1S,8aR)-2,5,5,8a-tetramethyl-4-oxo-4a,6,7,8-tetrahydro-1H-naphthalen-1-yl]-3-methylpentanoic acid	319,19	320,5	C**_20_** H**_32_** O**_3_**	12,24									Flavonoid
2-[(1S,2S,4aR,8aS)-1-hydroxy-4a-methyl-8-methylidene-1,2,3,4,5,6,7,8a-octahydronaphthalen-2-yl]prop-2-enoic acid	249,15	250,33	C**_15_** H**_22_** O**_3_**	10,98									Flavonoid
5,7-dihydroxy-2-(4-hydroxyphenyl)-8-[3,4,5-trihydroxy-6-(hydroxymethyl)oxan-2-yl]-6-(3,4,5-trihydroxyoxan-2-yl)chromen-4-one	563,16	564,5	C**_26_** H**_28_** O**_14_**	6,83	P(UT)	P(T)		P (T)			P(T)		Flavonoid
PG(22:6/0:0)	555,28	554,3	C**_26_** H**_51_** O**_10_** P	12,99	P(UT)		P(UT)		P(UT)	P(UT)	P(T)	P(UT)	Lipids
7-Glu tricin	491,12	492,4	C**_23_** H**_24_** O**_12_**	13,23	P (T)							P(T)	Flavonoid
16-hydroxypalmitic acid	271,23	272,42	C**_16_** H**_32_** O**_3_**	10,15									Fatty acid
[5-acetyloxy-3-(hydroxymethyl)-2-oxo-6-propan-2-ylcyclohex-3-en-1-yl] 3-methylpentanoate	339,14	340,4	C**_18_** H**_28_** O**_6_**	10,46									Flavonoid
(2R)-5,8-dihydroxy-2-(2-hydroxyphenyl)-7-methoxy-2,3-dihydrochromen-4-one	301,12	302,28	C**_16_** H**_14_** O**_6_**	3,07					P(T)				Lipids
PG(16:0/18:3)	744,48	745	C**_40_** H**_73_** O**_10_** P	13	P (T)			P(UT)	P(UT)				Lipids
9-KODE	293,18	294,5	C**_18_** H**_30_** O**_3_**	10,39			P (T)	P(UT)	P(UT)			P(T)	Fatty acid
Octyl-methoxycinnamate	291,16	290,4	C**_18_** H**_26_** O**_3_**	9,87									Carboxylic Acids
6,7-dimethoxy-2,2-dimethyl-2h-1-benzopyran	235,15	236,26	C**_13_** H**_16_** O**_4_**	11,55									Hormone
[5-acetyloxy-3-(hydroxymethyl)-2-oxo-6-propan-2-ylcyclohex-3-en-1-yl] ca	339,2	340,4	C**_18_** H**_28_** O**_6_**	12,21					P(T)				Carboxylic Acids
2-[(4-adamantanylphenyl)carbonylamino]-3-indol-3-ylpropanoic acid	441,26	442,6	C**_28_** H**_30_** N**_2_** O**_3_**	12,65									Fatty acids
2,4-dihydroxyheptadec-16-enyl acetate	327,25	328,5	C**_19_** H**_36_** O**_4_**	10,08				P(UT)	P(T)				Carboxylic Acids
Cyclopentaneacetic acid, 3-(hexopyranosyloxy)-2-[(2Z)-2-penten-1-yl]-	387,23	388,4	C**_18_** H**_28_** O**_9_**	12,54									Carboxylic Acids
Vitexin 2’’-O-rhamnoside II	593,15	594,5	C**_27_** H**_30_** O**_14_**	7,78									Flavonoid
Ellipticine	246,31	247,17	C**_17_** H**_14_** N**_2_**	11,49			P (T						Alkaloid
Silybin	481,14	482,44	C**_25_** H**_22_** O_10_	11,39					P (T)	P (T)			Flavonoid

*The P(T) shaded in grey represent the identified metabolite found in treated sample at 14- and 21- dpi and the P(UT) shaded in blue represent identified metabolite found in non-treated control sample at 14- and 21- dpi. K14dpi = Koonap at 14 days post inoculation, K21dpi = Koonap at 21 days post inoculation, M14dpi = Morocco at 14 days post inoculation, M21dpi = Morocco at 21 days post inoculation.

## 4 Discussion

Two virulent races of 2SA88 (TTKSF) and 2SA107 (PTKST) were inoculated on Koonap and Morocco at seedling stage in which no complete resistance ‘0’ was observed. The resistant wheat variety Koonap was noted having infection types ranging from ‘;’ to ‘2+’ for both races (**Figure 2**), indicating race-non-specific resistance in this variety. Previous research support this finding and have demonstrated that the variety Koonap confers long-lasting resistance to stem rust (Pretorius et al., 2000; Pretorius et al., 2007; Visser et al., 2011; Terefe et al., 2023). Our results of seedling tests against the two South African prevalent stem rust races also revealed that Koonap could be carrying effective seedling resistance metabolite biomarkers, as compared to Morocco where the vulnerability to Ug99 races on the variety was reported (Bajgain et al., 2016; Soko et al., 2018). The wheat variety Morocco is universally known to be relatively more susceptible to rust infection, which is in line with studies by Bender et al. (2016) that report on the large postules as indicators of the severity of the disease. The infection of the pathogen *P. graminis* seemingly spreads from 14 to 21 dpi for both races on the two varieties, indicating the disease progression between the two harvesting points. These findings provided further motivation to investigate the metabolites playing a role in *P. graminis* defence in the selected wheat varieties.

Quantified metabolic data can be explored for the determination of major metabolic differences between distinct wheat varieties as well as metabolic changes caused by *P. graminis* infection. In our study, the metabolomic profiles between the 2SA88 and 2SA107 infected wheat samples were almost similar (**Figure 1**), meaning that the genotype does not have a significant effect on the metabolite profile. The PCA model provided the virtual analysis of the effects of *P. graminis* treatments on wheat, mainly revealing clustering according to the time-points (**Figure 3**). This was indicative of the different metabolome profiles as the disease progressed (Mhlongo et al., 2020). The time-induced metabolomic changes following infection can be viewed in the context of metabolic pathways involved in the production of resistance related metabolites. The OPLS-DA score-plots further showed clear discrimination between the treated samples and control whereas S-plots allowed for the extraction of significant biomarkers (**Figure 4**). This observation is in agreement with previous works on wheat (Rudd et al., 2015; Cuperlovic-Culf et al., 2016; Duan et al., 2022; Mashabela et al., 2023), and other grains (Tugizimana et al., 2019; Li et al., 2022; Makhumbila et al., 2023). Overall, the separations observed gave insight into the chemistry and the differential metabolite profiles occurring in response to interactions with varying types of microbes. The biomarkers putatively annotated could serve in breeding programmes focusing on wheat improvement for rust resistance.

### 4.1 Role of annotated metabolites in defence against *P. graminis*

#### 4.1.1 Flavonoids

Plants produce a wide variety of low-molecular weight secondary metabolites called flavonoids that serve a wide range of purposes including signalling between plants and microbes (Groenbaek et al., 2019), as well as defensive roles against biotic stress (Sukumaran et al., 2018). In this study, the flavonoids vicenin-2, vicenin-3; 2-[(1S,2S,4aR,8aS)-1-hydroxy-4a-methyl-8-methylidene-1,2,3,4,5,6,7,8a-octahydronaphthalen-2-yl]prop-2-enoic acid; 7-Glu tricin; (2E)-3-[4-({2-O-[(2S,3R,4R)-3,4-Dihydroxy-4-(hydroxymethyl)tetrahydro-2-furanyl]-beta-D-glucopyranosyl}oxy)-3-methoxyphenyl]acrylic acid; vitexin-2-rhamnoside were detected, whereas flavonoids such as chrysoeriol; saponarin I and others were detected but not identified under different time-points induced by stem rust infection (**Table 2**). Our findings demonstrate that the cellular metabolome is reprogrammed as a result of the response to stem rust infection, evident by the dynamic and distinct changes in metabolite profiles. Following the OPLS-DA analysis in Koonap vs. Morocco, vitexin-2-rhamnoside, dirhamnosyl linolenic acid, 6,8-di-C-glucosyl apigenin, and saponarin I (majority of flavonoids) were positively correlated to Koonap and negatively correlated to Morocco. These results are in support of the study conducted by Mashabela et al. (2022), wherein the variety Koonap was also enriched with flavonoid glycosides. The metabolite vitexin and its derivatives is a natural flavonoid which is found in several plants including bamboo (Akter et al., 2021), mung bean (Wu et al., 2021), common buckwheat (Chua et al., 2020), hawthorn (Wu et al., 2021) and others. It has been shown to display antioxidant, anti-inflammatory, and anti-microbial effects (Noor et al., 2022). The other flavonoid glycosides detected in this study are shielding compounds that protect plants from oxidative damage caused by reactive oxygen species (ROSs) by slowing down oxidative degradation and scavenging free radicals (Groenbaek et al., 2019; Yang et al., 2020). In two separate studies reporting the metabolite profiling of wheat (Mashabela et al., 2022) and oat (Pretorius et al., 2022), varieties inoculated with *Puccinia triticina* and *Pseudomonas syringae* pv. Coronafaciens, respectively, elevated flavonoid glycosides production of dirhamnosyl linolenic acid, 6,8-di-C-glucosyl apigenin and others were also observed in response to inoculation with the two different plant pathogens. This is indicative of pathogen non-specificity of the detected flavonoid glycosides in protecting plants against diseases. A thorough dissection of the biosynthetic pathway and further investigation of the genes associated with the identified flavonoid glycosides could lead into the development of gene-based markers that can serve in breeding programs for rust resistance selection in wheat. Fractionation, isolation, purification and characterization of this group of biologically active secondary metabolites could also lead to a safer alternative to synthetic antifungal compounds or fungicides.

#### 4.1.2 Fatty acids and lipids

Pathogen defence in plants is significantly influenced by fatty acids (FAs) metabolic pathways (Kachroo and Kachroo, 2009; Perincherry et al., 2019). Recent research, however, shows that FAs and the by-products of their breakdown play more direct roles in stimulating different plant defence mechanisms (Zhu et al., 2018; Yan et al., 2020; Sharma et al., 2021). In this study, oxooctadecadienoic (9-OxoODE), hydroxyoctadecatrienoic acid (9-HOTrE) and trihydroxyoctadecanoic annotated from treated samples for both time-points, i.e., 14 dpi and 21 dpi, have been previously reported to possess phytotoxic or antifungal properties (Rawlinson et al., 2019; Nigro et al., 2020; Elshazly et al., 2022). An analysis of the concentration of these two fatty acids as the infection progresses (from early to late stages) could assist in understanding their specific role and durability in wheat rust resistance. Octadecatrienoyl-glycerol on the other hand, a lipid that contributes to the physical barrier of the cell wall (Žilić, 2016) was annotated only at 14dpi. This could mean that the metabolite gets suppressed with the progression of the infection.

#### 4.1.3 Carboxylic acids

Carboxylic acids or organic acids are compounds with a hydrocarbon radical with a carboxyl functional group connected to it (Kalgutkar and Daniels, 2010). These metabolites are often intermediating in amino acids-, lipids-, carbohydrates- and phenolic pathways (Xiao and Wu, 2014; Zhou et al., 2016). In order to produce molecules with a phenylpropane backbone under severe conditions, plants have exclusively acquired the ability to divert a significant quantity of carbon from the shikimic pathway into the different phenylpropanoid metabolism (Bhalla et al., 2005; Magazù et al., 2008; Vogt, 2010). The enzyme glucose-6-phosphate dehydrogenase (G6PDH), which transforms phosphate sugars to aromatic amino acids like phenylalanine, is mostly responsible for the formation of phenylpropanoids through the shikimic pathway. Later it is then used as the primary precursor and supplied into the phenylpropanoid biosynthesis pathway (Ali and Nozaki, 2007). In this current study, the carboxylic metabolites, cyclopentaneacetic acid, hydroxyamino, and dihydroxyheptadecane were differentially identified between the two wheat varieties, and thus could serve as biomarkers that distinguish between different genotypes (Rauf et al., 2021). The presence of these metabolites in the control and treated samples at both time-points is indicative of constitutive biosynthesis.

#### 4.1.4 Sugars

Sugar molecules function as nutrients as well as regulators of metabolism, growth, stress responses (Sheen et al., 1999; Smeekens, 2000; Rolland et al., 2002; Rolland and Sheen, 2005; Rolland et al., 2006; O’Hara et al., 2013), and membrane stability under a variety of abiotic and biotic conditions (Koch, 1996; Hoekstra et al., 2001; Rosa et al., 2009; Lemoine et al., 2013). In addition to serving as metabolic intermediates, photosynthates including glucose, sucrose, and certain of their derivatives also function as signalling molecules that affect the metabolism of plant cells. These sugars act as substrates for the production of fatty acids. It is known that the processes such as pathogen attack, wounding, and ultimately activation and repression of defence genes related to these processes are affected by glucose signalling through hexokinase dependent pathways (Tauzin and Giardina, 2014; Saddhe et al., 2021). Additionally, several studies where wheat was infected by a pathogen have demonstrated that *T. aestivum* may synthesize glucose and its glycosides as defence molecules (Brinkhaus et al., 2000; Satake and Kobuke, 2007; Shukri et al., 2011; Alqahtani et al., 2015). However, the present study did not find many of these compounds. Although hexopyranoside compound has been identified and biologically is categorized as sugar and was synthesised in the treated varieties at 21dpi (**Table 3**).

**Table 3:** Significant metabolic pathways found to be active in *P. graminis*, inferred from Metabolomics Pathway Analysis (MetPA).

Pathway Name	Match Status	p	-log(p)	Holm p	FDR	Impact	Details
Thiamine metabolism	2/22	0.027694	1.5576	1.0	1.0	0.11561	KEGG
Folate biosynthesis	2/27	0.040612	1.3913	1.0	1.0	0.08748	KEGG
Alpha-Linolenic acid metabolism	2/27	0.040612	1.3913	1.0	1.0	0.22025	KEGG
Glyoxylate and dicarboxylate metabolism	2/29	0.046315	1.3343	1.0	1.0	0.10147	KEGG
Riboflavin metabolism	1/11	0.12592	0.89991	1.0	1.0	0.06667	KEGG
Cutin, suberine and wax biosynthesis	1/14	0.15758	0.80251	1.0	1.0	0.25	KEGG
Sulfur metabolism	1/15	0.16789	0.77498	1.0	1.0	0.06077	KEGG
Purine metabolism	2/63	0.17544	0.75588	1.0	1.0	0.0	KEGG
Tyrosine metabolism	1/18	0.19811	0.70309	1.0	1.0	0.0	KEGG
Glycerolipid metabolism	1/21	0.2273	0.6434	1.0	1.0	0.11765	KEGG
Biosynthesis of unsaturated fatty acids	1/22	0.2368	0.62561	1.0	1.0	0.0	KEGG
Valine, leucine and isoleucine biosynthesis	1/22	0.2368	0.62561	1.0	1.0	0.02145	KEGG
Pyruvate metabolism	1/22	0.2368	0.62561	1.0	1.0	0.075	KEGG
Tryptophan metabolism	1/23	0.2462	0.60871	1.0	1.0	0.27586	KEGG
Cyanoamino acid metabolism	1/26	0.27373	0.56267	1.0	1.0	0.0	KEGG
Glycolysis / Gluconeogenesis	1/26	0.27373	0.56267	1.0	1.0	0.00151	KEGG
Galactose metabolism	1/27	0.2827	0.54867	1.0	1.0	0.0	KEGG
Glutathione metabolism	1/27	0.2827	0.54867	1.0	1.0	0.07071	KEGG
Terpenoid backbone biosynthesis	1/29	0.30032	0.52241	1.0	1.0	0.1008	KEGG
Glycine, serine and threonine metabolism	1/33	0.33435	0.4758	1.0	1.0	0.21178	KEGG

The Thiamine metabolism, Folate biosynthesis, Alpha-Linolenic acid metabolism and Glyoxylate and dicarboxylate metabolism pathways were the most impactful among the different cultivars and showed the highest statistical significance (p-value < 0.05).

#### 4.1.5 Nucleosides

G proteins, also referred to as guanine nucleotide-binding proteins, are a family of proteins that function as molecular switches within cells, conveying signals from a range of stimuli outside a cell to its interior (Gunnaiah and Kushalappa, 2014). A purine nucleoside such as guanosine, anchored on the cytoplasmic cell membrane were observed on the treated sample, Morocco at 14dpi and they are mediators for many cellular processes, including signal transduction, protein transport and growth regulation (Gogoi et al., 2001). According to Gunnaiah and Kushalappa (2014), G proteins are essential parts of plant defensive responses to pathogen challenge. Guanosine is a good signalling molecule that can reflect the metabolic and oxidative state of the cell due to its capacity to form complexes and its ease of metabolism in many cell compartments (Martínez-Reyes and Chandel, 2020). This finding suggested that guanosine and stem rust resistance may be tightly connected.

#### 4.1.6 Alkaloids

Commonly, alkaloids are concentrated in specific organs like the leaves, bark, or roots. It has been proposed that alkaloids in plants serve as a defence mechanism against pathogenic pests owing to their bitter taste (Kaur and Arora, 2015). Alkaloids act as shielding compounds that protect plants by scavenging free radicals and thereby reducing cell stress (Debegnach et al., 2019). These protective qualities can be a potent strategy in the fight against resistant microorganisms. A pyridoindole alkaloid known as ellipticine was identified in Koonap variety at 21 dpi. The source of pyrrolidine alkaloids is l-ornithine. L-ornithine is first converted to putrescine by the enzyme ornithine decarboxylase (ODC), which is subsequently converted to methylputrescine by the enzyme putrescine N-methyltransferase (Docimo et al., 2012). Methylputrescine is deaminated and oxidized to generate 1-methylpyrrolinium, the precursor of all pyrrolidine alkaloids, under the catalysis of primary amine: oxygen oxidoreductase (AOC).

### 4.2 Pathway analysis

**Figure 6:**
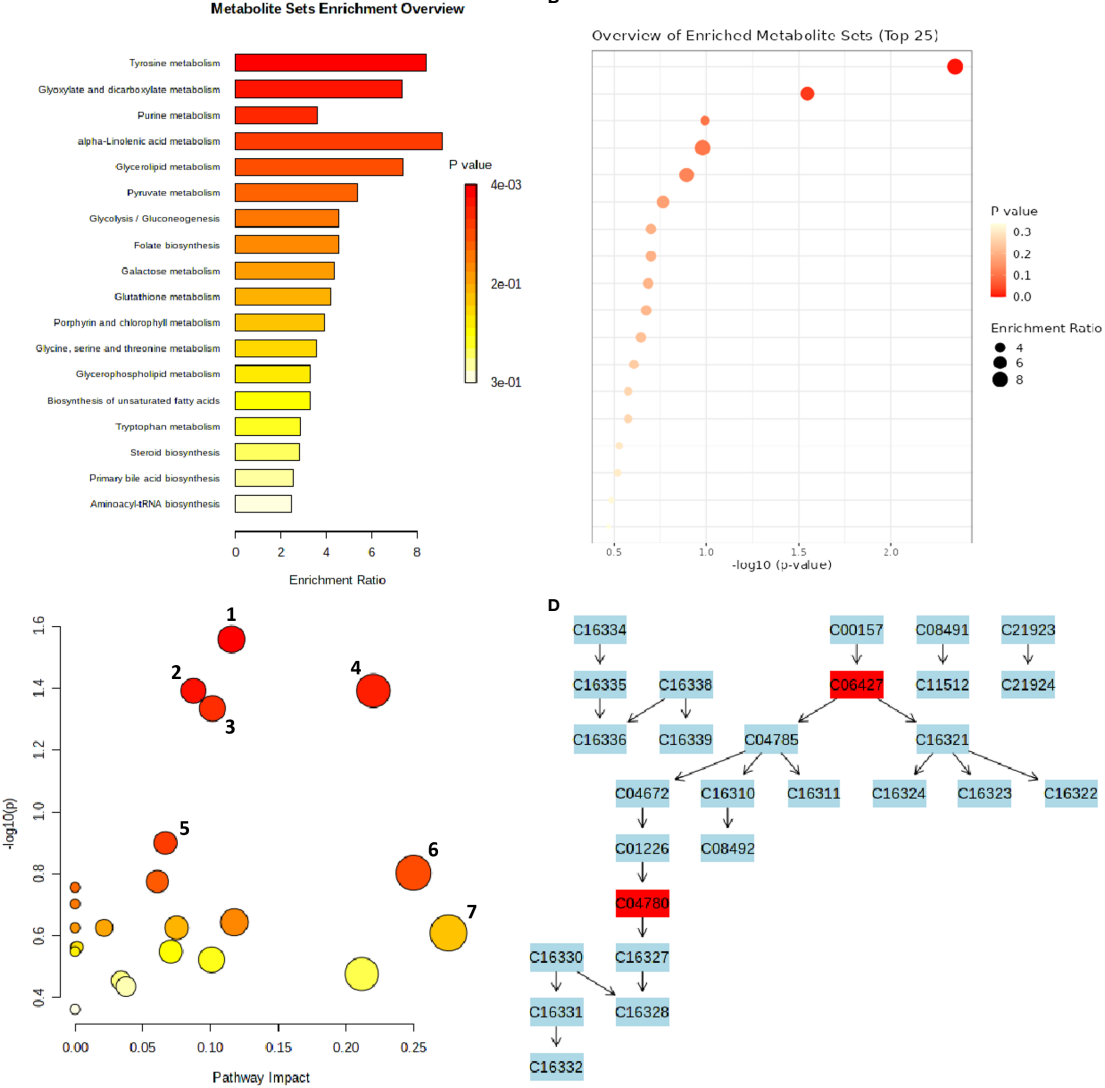
MetaboAnalyst-computed pathway analysis. **(A, B)** Representation of pathway enrichment analysis showing the metabolome view of the rust induced changes in different metabolic pathways. **(C)** Pathway view of statistically significant pathways flagged from the metabolome view based on matched metabolites, where the number represent 1) Thiamine pathway, 2) Folate biosynthesis, 3) Glyoxylate and dicarboxylate metabolism, 4) Alpha-Linolenic acid metabolism, 5) Riboflavin metabolism, 6) Cutin, suberine and wax biosynthesis, 7) Sulfur metabolism. **(D)** The diagram illustrates the integration and contribution of matched metabolites in the flagged Thiamine pathway.

An interactive visualization of pathway enrichment analysis at the metabolome level was presented in **Figures 6A, B**. Twenty biological pathways (**Table 3**) were identified by pathway enrichment analysis as having a substantial influence on stem rust tolerance. The pathways in **Figure 6C** are linked to the annotated metabolites of the two different wheat varieties. The KEGG pathway of the model plant *Oryza sativa* was used to map the discovered metabolites, illustrating the function of various metabolites in distinct metabolic pathways. Based on matched metabolites from the data, the thiamine; folate biosynthesis; alpha linolenic acid metabolism; and glyoxylate and dicarboxylate metabolism pathways exhibited relatively substantial influence. The riboflavin; cutin, suberin and wax biosynthesis; thiamine; folate; alpha linolenic metabolism; and glyoxylate and dicarboxylate metabolism pathways were the most statistically significant pathways.

Thiamine acts as a cofactor and activator to increase a plants’ resistance to disease and stress (Rapala-Kozik et al., 2012). According to the metabolic pathway, the metabolites were used as precursors for thiamine pyrophosphate (TPP), a crucial molecule required for metabolic processes such acetyl-CoA biosynthesis, amino acid biosynthesis, the Krebs cycle, and the Calvin cycle (Dong et al., 2016). The integration and contribution of matched metabolites in the highlighted thiamine pathway are shown in **Figure 6D**. The KEGG IDs are used to match the metabolites (e.g., orientin: C10195). The glyoxylate and dicarboxylate metabolism pathways, which result in the formation of oxoglutarate, share a metabolic pathway with the matching metabolites that serve as thiamine intermediates (**Figure 6D**) in the thiamine pathway (Nigro et al., 2020). The other top enriched pathways in this study; folate biosynthesis is a component of the methylation reaction required for the production of lipids, proteins, chlorophyll, and lignin (Klaus et al., 2005). One of the most significant metabolic routes in plants, alpha-linolenic serves as the main energy source for cellular metabolism. Riboflavin metabolism directs photosynthesis to the phenylpropanoid pathway, which is involved in critical processes such as plant development and growth as well as the reduction of biotic and abiotic stress (Boubakri et al., 2013). In addition, suberin, a glycerol-phenol-lipid polymer, plays an important role in the resistance/tolerance response of plants to external factors such as drought, salt, pests, and disease stress. It acts as an apoplastic barrier, by impeding pathogen invasion and minimising water and nutrient movement across the cell wall (Ranathunge et al., 2011; Holbein et al., 2019). Therefore, in resistant genotypes of wheat, it is evident that there’s enhanced accumulation of functional metabolic components such as thiamine, riboflavin, cutin, suberin, plant wax, and others, ensuring the normal growth and development of the plants.

## 5 Conclusion

The LC-qTOF-MS-based metabolomics approach revealed biomarkers associated with the wheat-pathogen interaction from four key classes of phenolic compounds such as flavonoids, hydroxycinnamic acid derivatives and polyphenols. The list also consists of fatty acids, carboxylic acids, and several sugars. In this study, it was discovered that the distributions of these metabolites varied depending on the differences between the two rust races and time-points. The findings further support the impact of differences based on a broader coverage of the metabolome and rich structural information from the interactions of the molecules in their natural environment. Overall, the riboflavin, cutin and suberin metabolism, thiamine, folate and alpha linolenic metabolism, and glyoxylate and dicarboxylate metabolism pathways were the most statistically significant pathways associated with the metabolites identified to be controlling wheat defence to *P. graminis*. Further investigations are however needed to better understand the mechanisms controlling these pathways, especially from the early to late stages of the infection. Moreover, studies on fractionation, isolation, purification and characterization of the detected biologically active group of secondary metabolites could lead to safer alternatives to synthetic antifungal compounds or fungicides. The potential for integrating metabolomics with other omics, such as genomics, transcriptomics, and proteomics, will aid in the discovery and characterization of significant candidate effectors in wheat pathogens at the molecular level and the development of novel molecular approaches for enhancing pathogen resistance in wheat. Additionally, crop breeders can use these tools to identify markers for desired traits, reintroduce genetic variation, and introduce desirable characteristics. The results from this study will pave a way to understand the mechanism of defence in wheat in response to rust pathogen infections.

## Data availability statement

The raw data supporting the conclusions of this article will be made available by the authors, without undue reservation.

## Author contributions

SF conceptualised and designed the experiment. MM, MR and SF conducted the plant growth experiments and applied the treatments. MM, MR and SF conducted the metabolite extractions. MM, MR, and EM conducted the LC-MS analysis. Data analysis was done by MM and EN. Revision of the manuscript was by MM, MR, EM, EN and SF. All authors contributed to the article and approved the submitted version.

## Supplementary material

Supplementary Figure 1 Representative UHPLC-QTOF-MS base peak intensity (BPI) chromatograms of Morocco cultivar methanolic-extracts. BPI MS chromatograms revealed differentially populated peaks for Morocco infected with rust race 2SA88 at 14-, 21 dpi (**A, B**, respectively), infected with rust race 2SA107 at 14-, 21 dpi (**C, D**, respectively) and control (**E, F**, respectively) each with unique m/z values, intensities and retention times (Rt’s), representing the qualitative (presence/absence) and quantitative (intensity/concentration) detection of metabolites, thus providing a visual description of the similarities and differences between the selected wheat varieties.

Supplementary Figure 2 OPLS-DA modelling showing the selection of signatory biomarkers associated with response of rust race infections of 2SA88 and 2SA107. The OPLS-DA scores plot **(A, C)** showed clear discrimination between the treated samples and control whereas S-plots **(B, D)** allowed for the extraction of significant biomarkers. Extracted ions representing significant biomarkers responsible for the difference between the control at the top right corner (in the blue rectangle) and samples treated with *Pgt.* at the bottom left corner (in the red rectangle) for both Koonap and Morocco cultivars at 21dpi, respectively. **Supplementary Figure 1** | The PCA score plot of *P. vulgaris* genotypes with varying resistance to *U. appendiculatus* races. **Supplementary Figure 2** | The OPLS-DA score plot of *P. vulgaris* genotypes with varying resistance to *U. appendiculatus* races.
