# Supplementary figures and images for: Defence-related metabolic changes in wheat (Triticum aestivum L.) seedlings in response to infection by Puccinia graminis f. sp. tritici
Source: Front Plant Sci. 2023 Jun 12;14:1166813. doi: 10.3389/fpls.2023.1166813 (PMC10292758; doi:10.3389/fpls.2023.1166813)

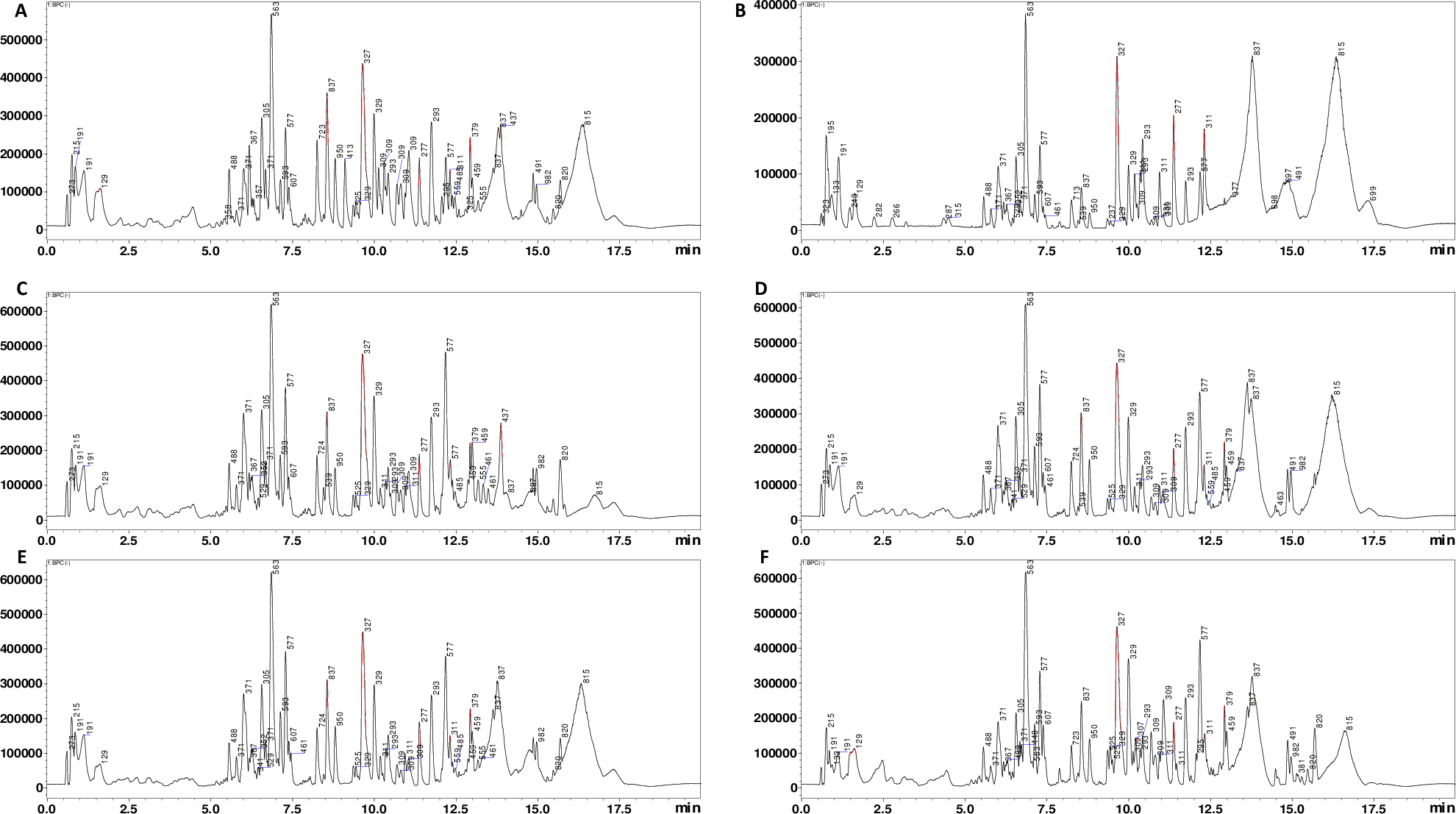

Supplement: Supplementary Figure 1 — Representative UHPLC-QTOF-MS base peak intensity (BPI) chromatograms of Morocco cultivar methanolic-extracts. BPI MS chromatograms revealed differentially populated peaks for Morocco infected with rust race 2SA88 at 14-, 21 dpi (A, B, respectively), infected with rust race 2SA107 at 14-, 21 dpi (C, D, respectively) and control (E, F, respectively) each with unique m/z values, intensities and retention times (Rt’s), representing the qualitative (presence/absence) and quantitative (intensity/concentration) detection of metabolites, thus providing a visual description of the similarities and differences between the selected wheat varieties. [file Image_1.tif]

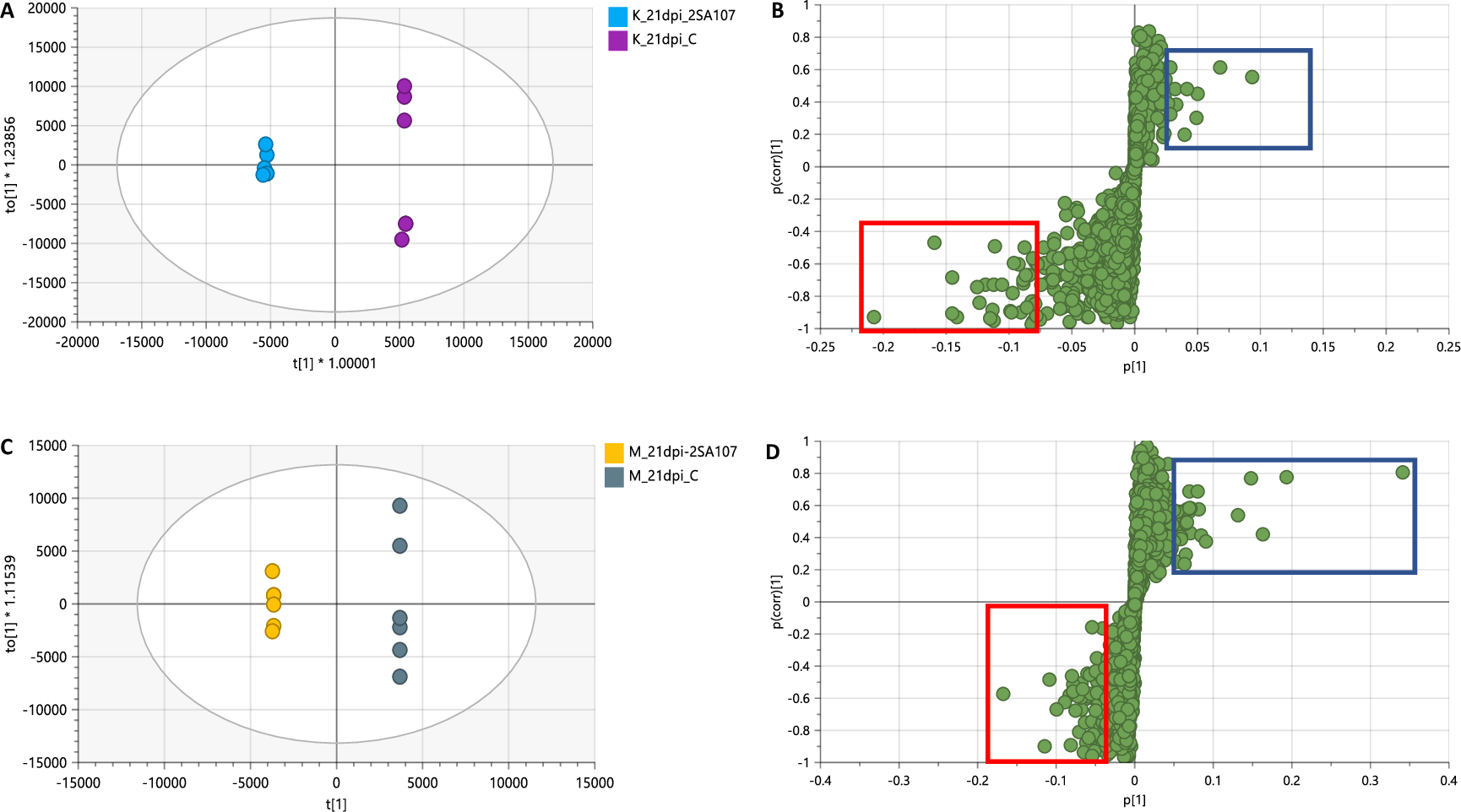

Supplement: Supplementary Figure 2 — OPLS-DA modelling showing the selection of signatory biomarkers associated with response of rust race infections of 2SA88 and 2SA107. The OPLS-DA scores plot (A, C) showed clear discrimination between the treated samples and control whereas S-plots (B, D) allowed for the extraction of significant biomarkers. Extracted ions representing significant biomarkers responsible for the difference between the control at the top right corner (in the blue rectangle) and samples treated with Pgt. at the bottom left corner (in the red rectangle) for both Koonap and Morocco cultivars at 21dpi, respectively. Supplementary Figure 1 | The PCA score plot of P. vulgaris genotypes with varying resistance to U. appendiculatus races. Supplementary Figure 2 | The OPLS-DA score plot of P. vulgaris genotypes with varying resistance to U. appendiculatus races. [file Image_2.tif]
